# Drosophila Keap1 Proteins Assemble Nuclear Condensates in Response to Oxidative Stress

**DOI:** 10.3390/antiox15010134

**Published:** 2026-01-21

**Authors:** Guangye Ji, Bethany Cross, Thomas Killmer, Bee Enders, Emma Neidviecky, Hayden Huber, Grace Lynch, Huai Deng

**Affiliations:** Department of Biology, University of Minnesota Duluth, Duluth, MN 55812, USA

**Keywords:** Keap1-Nrf2 oxidative response signaling, dKeap1, biomolecular condensates

## Abstract

The Keap1-Nrf2 signaling pathway is a central regulator of transcriptional responses to oxidative stress and is strongly linked to diverse pathologies, particularly cancer. In the cytoplasm, Keap1 (Kelch-like ECH-associated protein 1) promotes proteasomal degradation of Nrf2 (NF-E2–related factor 2). Oxidative stimuli disrupt the Keap1-Nrf2 interaction, facilitating Nrf2 nuclear accumulation and activation of antioxidant and detoxifying genes. Recent evidence suggests that Keap1 family proteins also enter the nucleus, bind chromatin, and regulate transcription, but the underlying mechanisms remain less understood. Here, we show that the *Drosophila* Keap1 ortholog, dKeap1, accumulates in the nucleus and gradually assembles stable nuclear foci in cells following oxidative treatment. FRAP analyses revealed reduced mobility of dKeap1 within these foci. Both the N-terminal (NTD) and C-terminal (CTD) domains of dKeap1 were required for foci formation. Two intrinsically disordered regions (IDRs) were identified within the CTD, and CTD-YFP fusion proteins readily formed condensates in vitro. Conversely, deletion of the Kelch domain resulted in robust cytoplasmic foci even under basal conditions, and in vitro assays also indicated that the Kelch domain suppresses dKeap1 condensate formation. Together, these findings reveal a novel molecular mechanism for the nuclear function of dKeap1, providing new insight into the broader roles of Keap1 factors in oxidative response, development, and disease.

## 1. Introduction

The Keap1-Nrf2 pathway plays a central role in cellular defense by mediating transcriptional responses against oxidative and xenobiotic stresses [1,2,3]. Dysregulation of this pathway contributes to many human diseases, including cancer, respiratory disorders, neurodegeneration, and cardiovascular disease [4,5,6,7,8]. Nrf2 (NF-E2–related factor 2) is a transcription factor that activates antioxidant and detoxification genes [4,9,10]. Under basal conditions, Keap1 (Kelch-like ECH-associated protein 1) interacts with Nrf2 in the cytoplasm and targets it for ubiquitin-dependent proteasomal degradation. Oxidative and xenobiotic stressors disrupt the Keap1-Nrf2 interaction, facilitating newly synthesized Nrf2 to enter the nucleus and activate its target genes [1,11].

Beyond stress responses, Keap1-Nrf2 signaling also regulates developmental programs in multiple organisms [12,13,14]. Keap1 and Nrf2 can directly target and regulate developmental transcripts, such as adipogenesis genes in mammals and *Drosophila* [15,16,17]. In *Drosophila*, CncC (cap-n-collar C) and dKeap1 (the orthologs of mammalian Nrf2 and Keap1, respectively) promote metamorphosis by activating ecdysone biosynthetic and response genes in specific tissues [14]. However, the molecular mechanisms by which Keap1 and Nrf2 proteins regulate developmental gene expression are not fully understood.

Interestingly, both mammalian Keap1 and *Drosophila* dKeap1 can localize to the nucleus [14,18,19]. Nuclear Keap1 was proposed to participate in the nuclear export of Nrf2 [20]. We previously demonstrated that dKeap1 can bind chromatin and function as a transcription activator in cooperation with CncC [14,19]. This nuclear function depends on the dKeap1 C-terminal domain, which mediates chromatin occupancy [15]. dKeap1 binds to the ecdysone-induced puffs on polytene chromosomes and activates ecdysone-response genes [14], while participate in gene silencing at pericentric heterochromatin [21], suggesting that dKeap1 controls developmental transcription likely through influencing chromatin structure. Consistently, dKeap1 physically and genetically interacts with B-type lamin and is required for maintaining normal nuclear lamina organization [22]. Together, these findings support a model in which nuclear dKeap1 regulates developmental transcription through chromatin remodeling mechanisms. Whether these Keap1 nuclear functions are linked to its canonical stress response roles remains to be explored.

A number of nuclear regulators assemble into biomolecular condensates, non-membranous compartments formed through liquid–liquid phase separation (LLPS) [23]. For example, Mediator and RNA polymerase II form condensates to control transcription initiation [24]. Formation of heterochromatin protein 1 α (HP1α) condensates can contribute to heterochromatin organization [25]. Polycomb repressive complex 1 (PRC1) forms chromatin-associated condensates via phase separation of CBX proteins, facilitating chromatin condensation and gene silencing [26,27]. Such condensates are typically scaffolded by proteins containing intrinsically disordered regions (IDRs), which can mediate phase separation through multiple ways of protein interactions [28].

To investigate the nuclear functions of Keap1 family proteins, we tracked the behavior of dKeap1 in live cells. Both YFP-tagged dKeap1 and endogenous dKeap1 formed distinct nuclear foci in response to oxidative stimuli. Formation of these foci requires the C-terminal domain, which contains two IDRs capable of driving condensate formation both in vivo and in vitro, whereas the Kelch domain of dKeap1 suppresses the formation of these foci. These findings reveal an unexpected capacity of dKeap1 to form biomolecular condensates and provide new insight into nuclear mechanisms by which Keap1 proteins may regulate chromatin structure and gene expression during stress responses.

## 2. Materials and Methods

### 2.1. Drosophila Stocks

Fly stocks were maintained at 25 °C according to standard protocol. Constructs of *UAS-YFP-dKeap1*, *UAS-YFP-dKeap1-∆NTD, UAS-YFP-dKeap1-∆Kelch*, or *UAS-YFP-dKeap1-∆CTD* were generated in the pUAST vector [29] and injected into the *w^1118^* background. *Sgs3-GAL4* and *tub-GAL4* [30] were from the Bloomington Stock Center.

### 2.2. Fluorescence Live Imaging

Salivary glands from L3 larvae, with or without oxidative treatment (0.5 mM H_2_O_2_ or 50 μM paraquat in PBS buffer), were mounted in 100 µL PBS on glass slides. A coverslip was placed on top with two edges raised using additional coverslips to prevent tissue compression. Samples were imaged, and movies were captured using a Nikon Eclipse Ni fluorescence microscope (Nikon USA, Melville, NY, USA) with 20× and 40× objectives.

### 2.3. Immunofluorescence

Salivary glands, fat tissue, gut, or brain complex isolated from L3 larvae are fixed in 3.7% paraformaldehyde for 5 min. Tissues were then washed with PBST (PBS + 0.2% Triton X-100) and stained with anti-GFP (NB600 1:200, Novus, Centennial, CO, USA), anti-lamin Dm0 (1:500, Developmental Studies Hybridoma Bank, Iowa City, IA, USA), dKeap1 antiserum (1:100) [14] and CncC antiserum (1:100) [14] at 4 °C overnight. After washing with PBST, the tissues were stained with 1:2000 goat anti-mouse Alexa Fluor 594 and goat anti-rabbit Alexa Fluor 488 secondary antibodies (Invitrogen, Carlsbad, CA, USA) at 25 °C for 2 h, washed with PBST, and then stained with Hoechst 33258 in PBS for 10 min. For the “smush” technique [31], salivary glands were gently compressed beneath a coverslip to release nuclei onto poly-lysine-coated slides before standard fixation and immunostaining as described above. Slides were mounted in Vectashield (Vector Laboratories, Newark, CA, USA) and imaged using a Nikon Eclipse Ti2 fluorescence microscope or a Nikon AX-R confocal microscope (Nikon USA, Melville, NY, USA). Detailed parameter settings for fluorescence imaging are described in the Supplemental Methods.

### 2.4. FRAP Assay

Salivary glands from L3 larvae, with or without oxidative treatment, were mounted in 100 µL PBS on glass slides. Imaging was conducted on a Nikon AX-R confocal microscope using a 40× objective and a 408 nm laser for photobleaching. The bleaching ROI was approximately 6 μm in diameter. Pre-bleach images were acquired every 6 s for three frames, followed by 20 s of bleaching. Recovery images were collected every 10 s for 2 min and then every 20 s for another 12 min. Fluorescence recoveries were quantified using Nikon NIS-Elements AR software (version 6.10.01). Intensities were corrected for background and overall photobleaching and normalized to pre-bleach levels. For each condition, 12 cells were analyzed from 6 independent salivary glands, and the normalized data were pooled to generate the mean recovery curve (± SD). Processed data were imported into R (version 4.5.1) for plotting. Half-recovery times (t½) were computed by linear interpolation at 50% of the recovery amplitude (plateau/2) using the normalized intensity values. FRAP recovery curves were generated using the ggplot2 package. A detailed protocol is provided in the Supplemental Methods.

### 2.5. Protein Purification

Full-length and truncated dKeap1 constructs fused with YFP were cloned into pGEX expression plasmids. *E. coli* BL21 competent cells were transformed with plasmids, grown to an OD600 of ~1.0, and induced with 0.5 mM IPTG for 3 h. GST-fusion proteins were purified from cell lysates using Glutathione Sepharose 4B (Cytiva, Marlborough, MA, USA) following standard protocol. GST tags were cleaved using PreScission Protease (APExBIO, Houston, TX, USA).

### 2.6. Phase Separation Assay

Purified proteins were added to condensate formation buffer (125 mM NaCl, 50 mM Tris-HCl, pH 7.4), without or with 10% PEG 8000, to result in final protein concentrations of 0.5 µM, 3.0 µM, and 5.0 µM. The mixtures were incubated at room temperature with gentle rocking for one hour. After the incubation, a 10 µL aliquot of solution was transferred onto a glass slide and allowed to settle for a few minutes before covering with a coverslip. Condensate formation, indicated by green fluorescence, was observed using a Nikon Eclipse Ti2 fluorescence microscope.

### 2.7. Statistical Analysis

All statistical analyses were performed in R (version 4.5.1) using custom scripts. For boxplot quantification, normality was assessed with the Shapiro–Wilk test, and homogeneity of variance was evaluated using Levene’s test. Depending on the data distribution, group comparisons were conducted using Welch’s one-way ANOVA, Mann–Whitney U test, or two-sided Wilcoxon rank-sum test. A significance threshold of *p* < 0.05 was applied for all tests.

## 3. Results

### 3.1. dKeap1 Proteins Form Nuclear Foci in Response to Oxidative Stimuli

**Figure 1:**
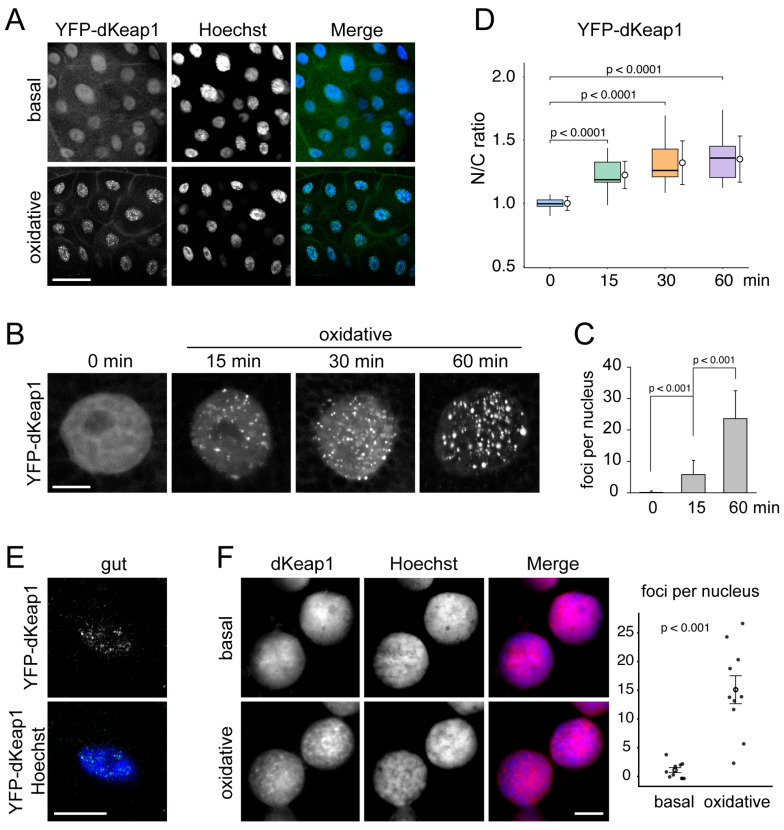
dKeap1 forms nuclear foci in response to oxidants: (**A**) YFP-dKeap1 forms nuclear foci in response to oxidative treatment. YFP-dKeap1 was expressed in salivary glands using the *Sgs3-GAL4* driver. Salivary gland fixed immediately (basal) or treated by 0.5 mM H_2_O_2_ for 30 min (oxidative) were stained with anti-GFP (green) and Hoechst for DNA (blue). Scale bar: 50 μm. (**B**) YFP-dKeap1 gradually forms foci upon oxidative stimulation. YFP-dKeap1 was visualized in salivary gland nuclei either without treatment (0 min) or after H_2_O_2_ exposure (oxidative) for the indicated times. Scale bar: 10 μm. (**C**) Quantification of YFP-dKeap1 foci formation. Distinct YFP-dKeap1 foci larger than 1 µm and exhibiting strong fluorescence intensity were counted in equatorial optical sections of salivary gland nuclei either without treatment (0 min) or after H_2_O_2_ exposure for the indicated times. Columns represent the mean number of nuclear foci, and error bars show the standard deviation calculated based on results from 30 cells of each group. Statistical significance was tested using one-way ANOVA. (**D**) Nuclear accumulation of YFP-dKeap1 after oxidative treatment. Fluorescence intensities of YFP-dKeap1 in nuclei and cytoplasm were measured, and nuclear-to-cytoplasmic (N/C) ratios were plotted. Box plots show the median and interquartile range, while circles indicate the mean value of each group. Standard deviations were calculated based on the results of 20 cells in each group. Statistical significance was tested using one-way ANOVA. (**E**) Oxidant-induced formation of dKeap1 foci in other tissues. YFP-dKeap1 was expressed using *tub-GAL4* driver. Cells from the gut were stained with anti-GFP (green) and Hoechst (blue). Scale bar: 10 μm. YFP-dKeap1 foci in fat and neuroblast cells are shown in Figure S1C,D. (**F**) Endogenous dKeap1 forms nuclear foci in response to oxidative stress. Wild-type salivary glands, either untreated (basal) or subjected to 60 min of oxidative stimulation, were prepared using the “smush” technique. Nuclei were stained with anti-dKeap1 (red) and Hoechst (blue). Scale bar: 10 μm. More nuclear staining images are shown in Figure S1E. Staining of the whole-mount tissues is shown in Figure S1F. Quantification of dKeap1 foci formation is shown on the right. Each dot represents an individual sample. Open circles indicate mean values, with error bars showing mean ± SEM. Statistical significance was determined using a two-sided Wilcoxon rank-sum test. Detailed methods for identifying and quantifying foci are described in the Supplemental Methods.

To investigate the behavior of dKeap1 in the living cell, we expressed YFP-dKeap1 in salivary gland cells using the *Sgs3-GAL4* driver [30]. YFP-dKeap1 localized to both the cytoplasm and nucleus but was enriched in the nucleus (Figure 1A,B), consistent with our previous observation [14,19]. Interestingly, when leaving the tissue in PBS for 15 min or longer, YFP-dKeap1 began to form multiple foci in the nucleus (Figure S1A).

We hypothesized that nuclear dKeap1 foci formation was triggered by oxidative stress. Supporting this idea, treatment of salivary glands with H_2_O_2_, which induces oxidative responses and activates Nrf2 signaling in cells [32], significantly accelerated the appearance of nuclear foci. Small foci emerged within 5 min of treatment, and their number and size increased over time (Figure 1A–C). Live imaging demonstrated that once formed, these foci were relatively stable: they moved only short distances within the nucleus and showed no fusion or fission events (Movies S1 and S2). Similar dKeap1 foci formation was observed upon the treatment of paraquat, another classic oxidant [33], which further supports that this foci formation is a consequence of oxidative stress (Figure S1B).

To determine whether oxidative stress also alters the subcellular distribution of dKeap1, we quantified nuclear and cytoplasmic fluorescence intensities of YFP-dKeap1. The nuclear-to-cytoplasmic (N/C) ratio increased significantly after oxidant exposure, indicating enhanced nuclear accumulation of dKeap1 (Figure 1D). These results suggest that oxidative stress triggers the translocation of dKeap1 from the cytoplasm into the nucleus.

These oxidant-induced YFP-dKeap1 foci were also detected in other cell types, including polyploid midgut and fat body cells (Figure 1E and Figure S1C), as well as diploid neuroblast cells (Figure S1C,D). Similarly, endogenous dKeap1, detected by immunostaining using a dKeap1-specific antibody identified previously [14], formed distinct nuclear foci following oxidative treatment (Figure 1F and Figure S1E,F).

### 3.2. YFP-dKeap1 Proteins Have Reduced Mobility in the Foci

**Figure 2:**
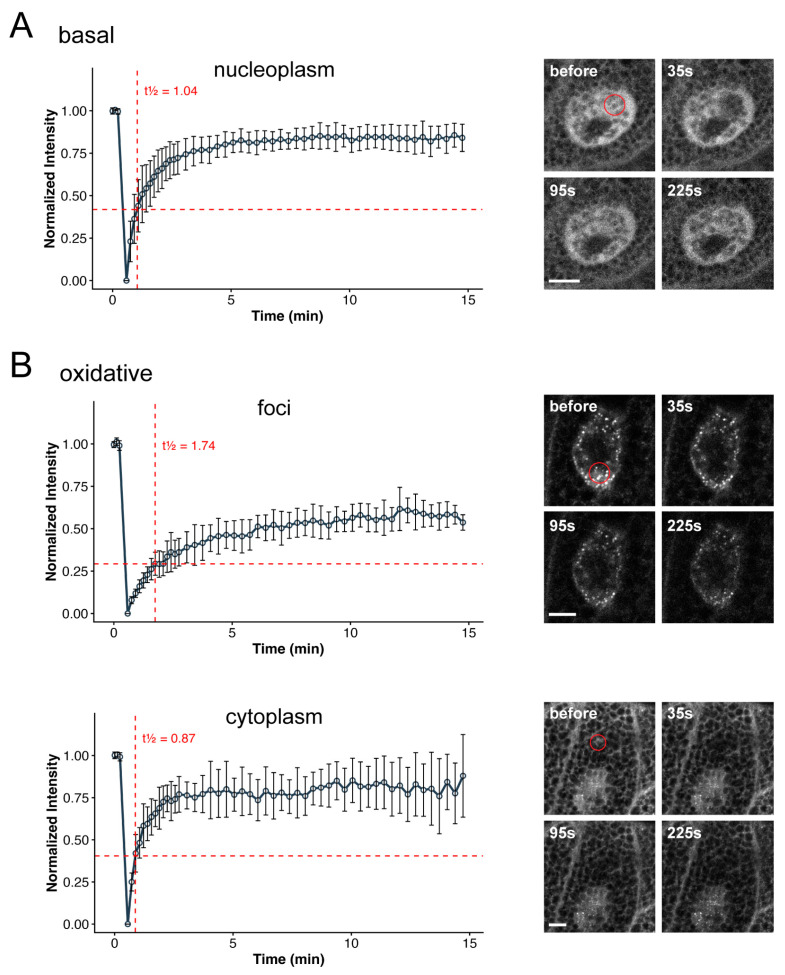
dKeap1 proteins exhibit reduced mobility in foci: (**A**) FRAP analysis of YFP-dKeap1 under basal conditions. Selective regions of salivary gland nuclei were photobleached, and fluorescence recovery was measured over time and plotted in a curve. Error bars indicate the standard deviation of 5 independent experiments. (**B**) FRAP analysis of YFP-dKeap1 under oxidative stress. Salivary glands were treated with H_2_O_2_ for 30 min prior to bleaching. Selective nuclear foci or cytoplasmic regions were bleached, and fluorescence recovery was recorded and plotted. Error bars indicate standard deviation from 5 independent experiments. Half-recovery times (t½) were calculated by linear interpolation at 50% of the recovery amplitude (horizonal red dash lines) using the normalized intensity values. Right: Representative images of a cell before and at the indicated time points after bleaching. The bleached region is highlighted by a red circle. Scale bars: 10 μm.

We next assessed the mobility of dKeap1 within these foci using the fluorescence recovery after photobleaching (FRAP) assay. Under basal conditions, nuclear YFP-dKeap1 recovered to approximately 80% of its pre-bleach intensity within 5 min and reached ~85% within 10 min, indicating high mobility (Figure 2A). In contrast, oxidant-induced nuclear YFP-dKeap1 foci recovered more slowly, reaching only ~60% within 10 min, suggesting substantially reduced mobility (Figure 2B, foci). Meanwhile, cytoplasmic YFP-dKeap1, which does not form foci under the same oxidative condition, recovered to around 80% within 5 min, comparable to the basal state (Figure 2B, cytoplasm). The half-time of recovery (t_1/2_) of YFP-dKeap1 was 0.87 s in the cytoplasm and 1.04 s in the nucleoplasm, but increased to 1.74 s within foci. These results indicate that dKeap1 exhibits lower mobility within foci and is likely composed of two molecular populations: a dynamic fraction and a more stable, less mobile fraction. dKeap1 outside foci remains highly dynamic, and its mobility appears unaffected by oxidative stress. Together, these findings suggest that the YFP-dKeap1 foci are not purely liquid droplets but may instead adopt gel-like condensate states [34].

### 3.3. Domain-Specific Regulation of dKeap1 Foci

**Figure 3:**
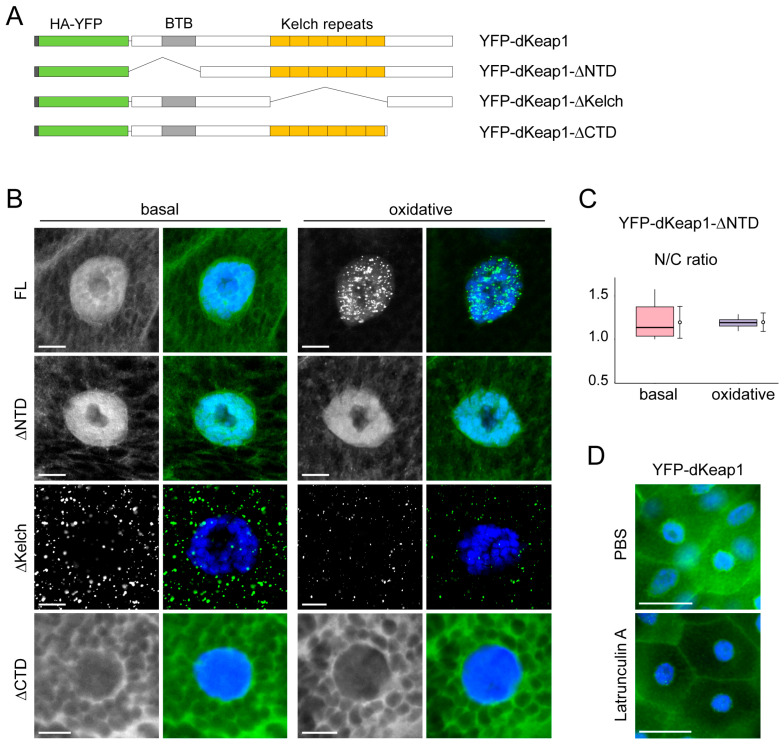
Function of dKeap1 domains in foci formation: (**A**) Schematic of dKeap1 fusion proteins. Full-length dKeap1 (FL) and truncated proteins lacking the N-terminal domain (ΔNTD), Kelch repeats (ΔKelch), or the C-terminal domain (ΔCTD) were tagged with HA and YFP. (**B**) Effects of domain deletions on dKeap1 foci formation. Salivary glands expressing the indicated dKeap1 fusion proteins were fixed immediately after dissection (basal) or following 60 min of H_2_O_2_ treatment (oxidative), and stained with anti-GFP (green) and Hoechst (blue). ΔNTD and ΔCTD variants failed to form oxidative stress-induced foci, whereas ΔKelch formed cytoplasmic foci independent of oxidative stimuli. Scale bar: 10 μm. (**C**) dKeap1-∆NTD does not accumulate in the nucleus upon oxidative treatment. Fluorescence intensities of YFP-dKeap1-ΔNTD in the nucleus versus cytoplasm were quantified, and nuclear-to-cytoplasmic (N/C) ratios are shown. Box plots indicate median and interquartile ranges, while circles represent mean values for each group. Standard deviations were calculated from 20 cells in each group. One-way ANOVA revealed no statistically significant difference between the two groups. (**D**) Actin filament disruption does not affect YFP-dKeap1 distribution. Salivary glands expressing YFP-dKeap1 were dissected and either fixed immediately (PBS) or treated with 1 µM latrunculin A for 20 min before fixation, followed by staining with anti-GFP (green) and Hoechst (blue). Additional results are shown in Figure S3A. Scale bar: 50 μm.

We next examined the contribution of dKeap1 domains to the formation of nuclear foci (Figure 3A). dKeap1 proteins depleting the N-terminus (ΔNTD) localized to both the nucleus and cytoplasm, with stronger nuclear accumulation, and oxidative stress did not alter its subcellular distribution or trigger foci formation (Figure 3B). These findings indicate that the NTD is required for stress-induced foci assembly. Furthermore, oxidative stress did not increase the nuclear abundance of the ΔNTD proteins (Figure 3C), suggesting that the NTD is also necessary for the nuclear enrichment of dKeap1. dKeap1 proteins depleting the C-terminus (ΔCTD) were retained in the cytoplasm and failed to form foci before or after oxidant treatment (Figure 3B). This result is consistent with our previous observation that the CTD is necessary for nuclear localization of dKeap1 [15] and further suggests a requirement for the CTD in nuclear foci formation.

Surprisingly, deletion of the Kelch repeats (ΔKelch) resulted in prominent cytoplasmic foci even under basal conditions, and oxidant treatment did not further alter this phenotype (Figure 3B). This suggests that the Kelch domain plays an inhibitory role in dKeap1 foci formation. Unlike YFP-dKeap1 foci, which exhibit limited movement within the nucleoplasm (Movies S1 and S2), the cytoplasmic foci formed by YFP-dKeap1-∆Kelch were stable and showed very low mobility (Movie S3). FRAP analysis revealed that dKeap1-∆Kelch proteins displayed reduced mobility in these cytoplastic foci, comparable to the mobility of dKeap1 in nuclear foci (Figure S2), suggesting that the microenvironments of these foci are similar. Together, these data demonstrate that both the NTD and CTD are required for dKeap1 condensate formation in cells, whereas the Kelch domain acts to prevent spontaneous condensate assembly.

Given that the Kelch repeats mediate Keap1 interaction with actin filaments [1], we hypothesized that binding to the actin network may prevent dKeap1 from forming foci in the cytoplasm. To test this possibility, we examined YFP-dKeap1 localization in salivary gland cells treated with Latrunculin A, an actin polymerization inhibitor [35]. In Latrunculin A-treated cells, some YFP-dKeap1 nuclear foci were observed, likely due to oxidative stress induced during the drug treatment (Figure 3D). However, no cytoplasmic foci were detected (Figure 3D and Figure S3A), indicating that actin binding is not the mechanism preventing dKeap1 foci formation in the cytoplasm.

### 3.4. dKeap1-CTD Carries IDRs and Forms Condensates In Vitro

**Figure 4:**
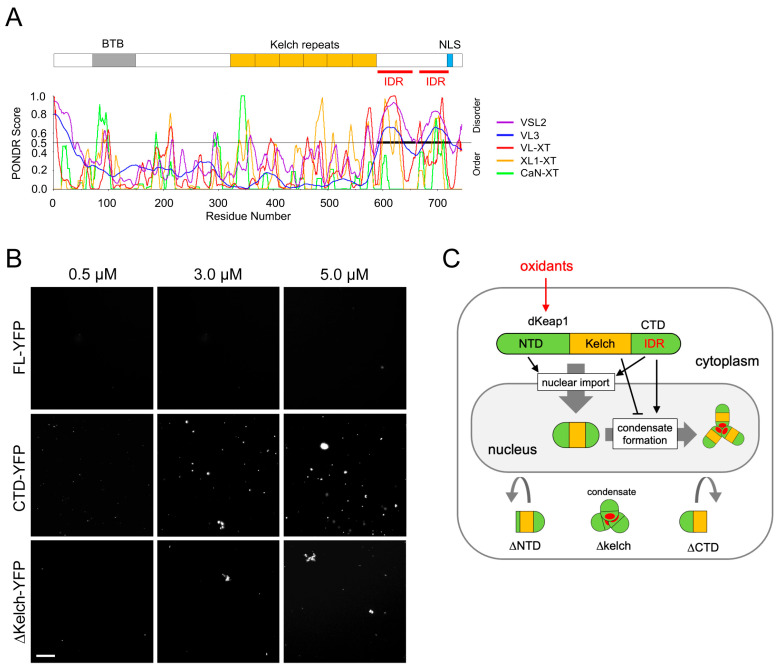
dKeap1-CTD drives condensate formation in vitro: (**A**) Prediction of IDRs in the C-terminus of dKeap1. The amino acid sequence of dKeap1 was analyzed using PONDR, which identified two adjacent intrinsically disordered regions (red lines) encompassing ~80% of the C-terminal domain. (**B**) In vitro condensate formation by dKeap1 variants. dKeap1 full length (FL), CTD, or ∆Kelch fused with YFP at the C-terminus were purified. Different concentrations of these proteins indicated above were incubated with 10% PEG 8000 crowding agent. Green fluorescence was imaged, and representative regions are shown. Full-length dKeap1 did not form condensates, whereas both the CTD and the Kelch-depleting truncation formed condensates in vitro. Scale bar: 10 μm. (**C**) Model of nuclear dKeap1 condensate formation. Oxidative stimuli promote nuclear entry of dKeap1 and drive the formation of dKeap1 condensates within the nucleus. Both the NTD and CTD are required for nuclear transport, whereas IDRs within the CTD mediate condensate formation. In contrast, the Kelch domain prevents condensate formation in the basal condition.

The nuclear foci formed by YFP-dKeap1 are likely biomolecular condensates. Using the Predictor of Natural Disordered Regions (PONDR) [36], we identified two intrinsic disorder regions (IDRs) within the CTD of dKeap1 (Figure 4A), suggesting a potential for phase separation-driven condensate formation. To test this hypothesis, we generated and purified dKeap1-YFP, dKeap1-CTD-YFP, and dKeap1-ΔKelch-YFP fusion proteins and performed in vitro phase separation assays. dKeap1-CTD-YFP robustly formed condensates in a concentration-dependent manner (Figure 4B). In contrast, full-length dKeap1-YFP (FL-YFP) did not phase separate (Figure 4B), consistent with our in vivo findings that YFP-dKeap1 does not spontaneously form foci under basal conditions (Figure 1A and Figure 3B). Notably, dKeap1-ΔKelch-YFP formed condensates in vitro (Figure 4B), aligning with the observation that YFP-dKeap1-ΔKelch forms cytoplasmic foci in cells even without oxidative stress. It is also notable that the efficiency of dKeap1-ΔKelch-YFP condensate formation was lower than that of CTD-YFP, indicating that other regions of dKeap1 influence but do not block CTD-mediated phase separation.

We repeated the in vitro assay in buffer without the crowding reagent PEG (Figure S4). Consistently, YFP-dKeap1 did not form condensates, whereas condensates formed by CTD-YFP and YFP-dKeap1-ΔKelch were still observed, although their number was noticeably reduced compared with PEG-containing conditions. This indicates that the crowding reagent is not required for LLPS and condensate formation but enhances droplet abundance. Taken together, these results support a model in which IDRs in the CTD drive phase separation and condensate formation of dKeap1, while the Kelch domain inhibits condensate formation (Figure 4C).

### 3.5. CncC and Lamin Are Not Associated with dKeap1 Foci

dKeap1 interacts with CncC in both the cytoplasm and nucleus [19]. To determine whether dKeap1 condensates can recruit CncC, we examined CncC localization in cells overexpressing YFP-dKeap1. As expected, YFP-dKeap1 overexpression markedly reduced endogenous CncC levels, consistent with the inhibitory role of Keap1 proteins toward Nrf2/CncC. CncC immunosignals were largely absent from the nucleus, and no detectable CncC signal colocalized with dKeap1 foci. Our current data do not support that dKeap1 condensates serve as a major site for dKeap1-CncC complex formation.

Our recent study showed that dKeap1 interacts with *Drosophila* B-type lamin (lamin Dm0), and that overexpression of YFP-dKeap1 induces relocalization of lamin Dm0 from the nuclear periphery to the nucleoplasm [22]. We therefore examined whether lamin Dm0 is associated with YFP-dKeap1 condensates. Consistent with our previous findings, YFP-dKeap1 expression in salivary gland cells caused ectopic localization of lamin Dm0 in the nucleoplasm, where some obvious lamin foci were present (Figure S3B). However, these lamin foci showed minimal colocalization with YFP-dKeap1 foci (Figure S3B). Thus, YFP-dKeap1 condensates do not appear to host lamin Dm0 and are likely not directly responsible for the dKeap1-induced lamin mislocalization phenotype.

## 4. Discussion

In contrast to the well-established cytoplasmic role of Keap1 as an adaptor protein in a Cullin-3 E3 ubiquitin ligase complex that promotes proteasomal degradation of Nrf2 and thereby suppresses Nrf2-mediated antioxidant and detoxifying transcripts, the functions of nuclear Keap1 remain much less understood. In mammalian cells, Keap1 has been reported to shuttle into the nucleus and facilitate the nuclear export of Nrf2 [18,20]. In *Drosophila*, a substantial pool of dKeap1 localizes within the nucleus. We have shown that nuclear dKeap1 associates with chromatin, where it contributes to transcriptional activation of developmental genes and, on the other hand, establishment of pericentric heterochromatin [14,15,19,21]. However, a major fraction of nuclear dKeap1 resides in the nucleoplasm rather than binding to chromatin, and the functions of this dKeap1 population remain unclear. In this study, we found that dKeap1 can form condensates in the nucleoplasm in response to oxidative stress.

Our previous work showed that xenobiotic stimuli can increase dKeap1 occupancy at specific genomic loci [19]. Here, we extend this model by demonstrating that oxidative stress enhances the overall nuclear abundance of dKeap1. We further show that oxidative stress induces dKeap1 to form nuclear condensates (Figure 4C). The spherical morphology and mobility of these foci suggest that they reside in the nucleoplasm rather than on chromatin, as proteins and complexes binding to the polytene chromosome, large structures formed by the alignment of more than a thousand interphase chromatids in polyploid cells, typically form immobile band-like patterns [19,31]. This discovery suggests that oxidative stress can not only regulate Keap1’s canonical cytoplasmic function but also likely controls its nuclear functions.

The reduced mobility of dKeap1 in these nuclear foci, as revealed by the FRAP assay, indicates that these foci are not purely liquid droplets but may instead adopt gel-like condensate states. Many biomolecular condensates in cells are known to span a spectrum of states ranging from liquid-like to gel-like or even solid-like [34]. For example, nuclear PRC1 condensates and HP1 condensates exhibit low FRAP recovery and display gel-like properties, which may allow proteins to be retained in a semi-stable state while maintaining relatively stable yet dynamic chromatin and gene regulation [37,38]. We therefore hypothesize that dKeap1 foci may be initiated through LLPS and subsequently mature into gel-like condensates, potentially driven by protein structural rearrangements, protein-protein interactions, or post-translational modifications.

Most of the results described in this study were obtained using YFP-dKeap1 fusion proteins overexpressed in *Drosophila* tissues via the UAS-GAL4 system. The expression levels of YFP-dKeap1 fusions are approximately 100-fold higher than endogenous dKeap1 [14,19], which could introduce some artifacts, such as stronger foci signals. However, we believe these results largely reflect the native mechanism for two reasons. First, dKeap1 immunostaining shows that endogenous dKeap1 also forms foci with a similar pattern, although with weaker signal intensity (Figure 1F and Figure S1E,F). Second, different dKeap1 truncations significantly affect foci formation (Figure 3B), indicating that this phenotype is not merely a result of YFP-fusion overexpression but is more likely a regulatable mechanism in the cell.

We found that both nuclear accumulation and condensate formation of dKeap1 require its N-terminal region. The N-terminus of dKeap1 contains a BTB domain, which mediates dKeap1 dimerization and interaction with the E3 complex [4]. However, because dKeap1 dimers are found predominantly in the cytoplasm [19], our data indicate that dKeap1 dimerization is not required for the formation of nuclear condensates.

The C-terminal domain (CTD) of dKeap1 is known to mediate nuclear localization and chromatin binding [15]. Our in vivo and in vitro results now indicate that the CTD is also necessary for dKeap1 condensate formation. The discovery of intrinsically disordered regions (IDRs) within the CTD supports a model in which this region drives phase separation and condensate assembly. In support of this model, our FRAP assays revealed that the mobility of dKeap1 is markedly reduced within the nuclear foci and that a fraction of dKeap1 is immobile. Such reduced mobility and the presence of an immobile population are common features of proteins within biomolecular condensates [39,40], since IDRs mediate multivalent interactions among proteins.

Interestingly, although the CTD alone exhibits phase-separation capacity in vitro, full-length dKeap1 does not spontaneously form condensates under basal conditions. We found that the Kelch domain suppresses condensate formation, as its removal resulted in robust focus formation both in cells and in vitro. This inhibitory effect does not appear to be mediated through actin binding, a known function of the Kelch repeats. Instead, we speculate that the Kelch domain constrains dKeap1 in a conformation that prevents phase separation in the basal condition, and oxidative stimuli probably induce conformational changes in dKeap1 that relieve this inhibition.

Oxidants and xenobiotic electrophiles modify key cysteine residues in Keap1, altering protein conformation and interfering with its interaction with Nrf2 [4,11,41,42]. Our findings indicate a hypothesis in which oxidative modification of residues in multiple regions of dKeap1, particularly within the Kelch domain, relieves an inhibitory mechanism that normally prevents the formation of nuclear condensates. Several cysteine residues (C434, C489, and C583) in the Kelch domain are evolutionarily conserved [4,33], raising the possibility that their oxidative modification alters dKeap1 conformation in a way that enables the CTD to undergo phase separation. Because condensates form exclusively in the nucleus, there might be additional spatially restricted factors to either prevent condensate formation in the cytoplasm or facilitate their assembly within the nuclear environment.

The functional significance of dKeap1 nuclear condensates remains unclear. dKeap1 can form a complex with CncC on chromatin and function as a transcriptional coactivator [19]. Nuclear dKeap1 also physically interacts with B-type lamin, and elevated dKeap1 levels can alter lamin Dm0 organization [22]. However, our current results do not support the recruitment of either CncC or lamin into dKeap1 condensates. In the cytoplasm, Keap1 functions as an adaptor in the Cul3-Rbx1 E3 ubiquitin ligase complex [1]. Several nuclear E3 ligase complexes have been identified that regulate chromatin structure, DNA repair, and mitosis, and some assemble into condensates. For example, the PRC1 complex, which contains the RING1B E3 ligase, forms nuclear condensates mediated by phase separation of CBX2 [40]. Additional E3 complexes, such as Cullin-4 [43] and APC/C [44], also exist in the nucleus and are conserved in *Drosophila*. Whether dKeap1 participates in a nuclear E3 complex and whether such complexes are recruited to dKeap1 condensates remain open questions.

Given the recently revealed functions of dKeap1 in the activation of developmental genes [14,15], it will be interesting to assess whether dKeap1 condensates play a role in regulating transcription. Several developmental regulators that control nuclear architecture and transcription, such as the Mediator and the PRC1 complex [24,27], form condensates via LLPS. It has also been shown that Oct4 can regulate stem cell fate by forming condensates and reorganizing TADs [45]. In *Drosophila*, in vitro assays indicate that the PRC1 component Ph undergoes LLPS with chromatin [46], and *Drosophila* insulator proteins, known regulators of nuclear architecture and developmental transcription, exhibit LLPS in S2 cells [47]. Taken together, identifying the molecular components and biological functions of the dKeap1 condensates will be an important direction for future studies.

Keap1-family proteins are central cellular sensors of xenobiotic and oxidative stress, and the Keap1-Nrf2 pathway plays key roles in both stress responses and developmental regulation. A deeper understanding of nuclear Keap1 mechanisms will broaden our knowledge of how environmental toxins influence development and physiology and may help reveal novel mechanisms of Keap1/Nrf2 in human disease.

## Data Availability

The original contributions presented in this study are included in the article/Supplementary Material. Further inquiries can be directed to the corresponding authors.

## Supplementary Materials

The following supporting information can be downloaded at: https://www.mdpi.com/article/10.3390/antiox15010134/s1. Figure S1: dKeap1 forms nuclear foci in response to oxidative stresses; Figure S2: dKeap1-∆Kelch exhibits reduced mobility in cytoplasmic foci; Figure S3: Actin filament and lamin are not associated with dKeap1 condensates; Figure S4: dKeap1-CTD undergoes LLPS in vitro; Movies S1–S3: Dynamics of YFP-dKeap1 foci in salivary gland cells; Supplemental Materials and Methods.

## References

[1] Itoh K., Wakabayashi N., Katoh Y., Ishii T., Igarashi K., Engel J.D., Yamamoto M. (1999). Keap1 Represses Nuclear Activation of Antioxidant Responsive Elements by Nrf2 through Binding to the Amino-Terminal Neh2 Domain. Genes Dev..

[2] Slocum S.L., Kensler T.W. (2011). Nrf2: Control of Sensitivity to Carcinogens. Arch. Toxicol..

[3] Wakabayashi N., Itoh K., Wakabayashi J., Motohashi H., Noda S., Takahashi S., Imakado S., Kotsuji T., Otsuka F., Roop D.R. (2003). Keap1-Null Mutation Leads to Postnatal Lethality Due to Constitutive Nrf2 Activation. Nat. Genet..

[4] Taguchi K., Motohashi H., Yamamoto M. (2011). Molecular Mechanisms of the Keap1–Nrf2 Pathway in Stress Response and Cancer Evolution. Genes Cells Devoted Mol. Cell. Mech..

[5] Carlson J., Price L., Deng H. (2020). Nrf2 and the Nrf2-Interacting Network in Respiratory Inflammation and Diseases. Nrf2 Its Modul. Inflamm..

[6] Smith R.E., Tran K., Smith C.C., McDonald M., Shejwalkar P., Hara K. (2016). The Role of the Nrf2/ARE Antioxidant System in Preventing Cardiovascular Diseases. Diseases.

[7] Uruno A., Matsumaru D., Ryoke R., Saito R., Kadoguchi S., Saigusa D., Saito T., Saido T.C., Kawashima R., Yamamoto M. (2020). Nrf2 Suppresses Oxidative Stress and Inflammation in App Knock-In Alzheimer’s Disease Model Mice. Mol. Cell. Biol..

[8] Sykiotis G.P., Bohmann D. (2010). Stress-Activated Cap’n’collar Transcription Factors in Aging and Human Disease. Sci. Signal..

[9] Malhotra D., Portales-Casamar E., Singh A., Srivastava S., Arenillas D., Happel C., Shyr C., Wakabayashi N., Kensler T.W., Wasserman W.W. (2010). Global Mapping of Binding Sites for Nrf2 Identifies Novel Targets in Cell Survival Response Through ChIP-Seq Profiling and Network Analysis. Nucleic Acids Res..

[10] Zhang D.D. (2006). Mechanistic Studies of the Nrf2-Keap1 Signaling Pathway. Drug Metab. Rev..

[11] Eggler A.L., Liu G., Pezzuto J.M., van Breemen R.B., Mesecar A.D. (2005). Modifying Specific Cysteines of the Electrophile-Sensing Human Keap1 Protein Is Insufficient to Disrupt Binding to the Nrf2 Domain Neh2. Proc. Natl. Acad. Sci. USA.

[12] Pitoniak A., Bohmann D. (2015). Mechanisms and Functions of Nrf2 Signaling in Drosophila. Free Radic. Biol. Med..

[13] Morgenstern C., Lastres-Becker I., Demirdöğen B.C., Costa V.M., Daiber A., Foresti R., Motterlini R., Kalyoncu S., Arioz B.I., Genc S. (2024). Biomarkers of NRF2 Signalling: Current Status and Future Challenges. Redox Biol..

[14] Deng H., Kerppola T.K. (2013). Regulation of Drosophila Metamorphosis by Xenobiotic Response Regulators. PLoS Genet..

[15] Carlson J., Price L., Cook I., Deng H. (2022). Drosophila Keap1 Xenobiotic Response Factor Regulates Developmental Transcription Through Binding to Chromatin. Dev. Biol..

[16] Huang J., Tabbi-Anneni I., Gunda V., Wang L. (2010). Transcription Factor Nrf2 Regulates SHP and Lipogenic Gene Expression in Hepatic Lipid Metabolism. Am. J. Physiol. Gastrointest. Liver Physiol..

[17] Pi J., Leung L., Xue P., Wang W., Hou Y., Liu D., Yehuda-Shnaidman E., Lee C., Lau J., Kurtz T.W. (2010). Deficiency in the Nuclear Factor E2-Related Factor-2 Transcription Factor Results in Impaired Adipogenesis and Protects against Diet-Induced Obesity. J. Biol. Chem..

[18] Sun Z., Wu T., Zhao F., Lau A., Birch C.M., Zhang D.D. (2011). KPNA6 (Importin A7)-Mediated Nuclear Import of Keap1 Represses the Nrf2-Dependent Antioxidant Response. Mol. Cell. Biol..

[19] Deng H., Kerppola T.K. (2014). Visualization of the Drosophila dKeap1-CncC Interaction on Chromatin Illumines Cooperative, Xenobiotic-Specific Gene Activation. Development.

[20] Velichkova M., Hasson T. (2005). Keap1 Regulates the Oxidation-Sensitive Shuttling of Nrf2 into and out of the Nucleus via a Crm1-Dependent Nuclear Export Mechanism. Mol. Cell. Biol..

[21] Carlson J., Swisse T., Smith C., Deng H. (2019). Regulation of Position Effect Variegation at Pericentric Heterochromatin by Drosophila Keap1-Nrf2 Xenobiotic Response Factors. Genesis.

[22] Carlson J., Neidviecky E., Cook I., Cross B., Deng H. (2024). Interaction with B-Type Lamin Reveals the Function of Drosophila Keap1 Xenobiotic Response Factor in Nuclear Architecture. Mol. Biol. Rep..

[23] Banani S.F., Lee H.O., Hyman A.A., Rosen M.K. (2017). Biomolecular Condensates: Organizers of Cellular Biochemistry. Nat. Rev. Mol. Cell Biol..

[24] Cho W.-K., Spille J.-H., Hecht M., Lee C., Li C., Grube V., Cisse I.I. (2018). Mediator and RNA Polymerase II Clusters Associate in Transcription-Dependent Condensates. Science.

[25] Strom A.R., Emelyanov A.V., Mir M., Fyodorov D.V., Darzacq X., Karpen G.H. (2017). Phase Separation Drives Heterochromatin Domain Formation. Nature.

[26] Tatavosian R., Kent S., Brown K., Yao T., Duc H.N., Huynh T.N., Zhen C.Y., Ma B., Wang H., Ren X. (2019). Nuclear Condensates of the Polycomb Protein Chromobox 2 (CBX2) Assemble through Phase Separation. J. Biol. Chem..

[27] Brown K., Chew P.Y., Ingersoll S., Espinosa J.R., Aguirre A., Espinoza A., Wen J., Astatike K., Kutateladze T.G., Collepardo-Guevara R. (2023). Principles of Assembly and Regulation of Condensates of Polycomb Repressive Complex 1 through Phase Separation. Cell Rep..

[28] Holehouse A.S., Kragelund B.B. (2024). The Molecular Basis for Cellular Function of Intrinsically Disordered Protein Regions. Nat. Rev. Mol. Cell Biol..

[29] Brand A.H., Perrimon N. (1993). Targeted Gene Expression as a Means of Altering Cell Fates and Generating Dominant Phenotypes. Development.

[30] Cherbas L., Hu X., Zhimulev I., Belyaeva E., Cherbas P. (2003). EcR Isoforms in Drosophila: Testing Tissue-Specific Requirements by Targeted Blockade and Rescue. Development.

[31] Johansen K.M., Cai W., Deng H., Bao X., Zhang W., Girton J., Johansen J. (2009). Polytene Chromosome Squash Methods for Studying Transcription and Epigenetic Chromatin Modification in Drosophila Using Antibodies. Methods.

[32] Fourquet S., Guerois R., Biard D., Toledano M.B. (2010). Activation of NRF2 by Nitrosative Agents and H_2_O_2_ Involves KEAP1 Disulfide Formation. J. Biol. Chem..

[33] Sykiotis G.P., Bohmann D. (2008). Keap1/Nrf2 Signaling Regulates Oxidative Stress Tolerance and Lifespan in Drosophila. Dev. Cell.

[34] Choi S., Lee J.-M., Kim K.K. (2025). Biomolecular Condensates: Molecular Structure, Biological Functions, Diseases, and Therapeutic Targets. Mol. Biomed..

[35] Knoblich J.A., Jan L.Y., Jan Y.N. (1997). The N Terminus of the Drosophila Numb Protein Directs Membrane Association and Actin-Dependent Asymmetric Localization. Proc. Natl. Acad. Sci. USA.

[36] Peng K., Radivojac P., Vucetic S., Dunker A.K., Obradovic Z. (2006). Length-Dependent Prediction of Protein Intrinsic Disorder. BMC Bioinform..

[37] Seif E., Francis N.J. (2024). A Two-Step Mechanism for Creating Stable, Condensed Chromatin with the Polycomb Complex PRC1. Molecules.

[38] Ackermann B.E., Debelouchina G.T. (2019). Heterochromatin Protein HP1α Gelation Dynamics Revealed by Solid-State NMR Spectroscopy. Angew. Chem. Int. Ed..

[39] Larson A.G., Elnatan D., Keenen M.M., Trnka M.J., Johnston J.B., Burlingame A.L., Agard D.A., Redding S., Narlikar G.J. (2017). Liquid Droplet Formation by HP1α Suggests a Role for Phase Separation in Heterochromatin. Nature.

[40] Plys A.J., Davis C.P., Kim J., Rizki G., Keenen M.M., Marr S.K., Kingston R.E. (2019). Phase Separation of Polycomb-Repressive Complex 1 Is Governed by a Charged Disordered Region of CBX2. Genes Dev..

[41] Yamamoto M., Kensler T.W., Motohashi H. (2018). The KEAP1-NRF2 System: A Thiol-Based Sensor-Effector Apparatus for Maintaining Redox Homeostasis. Physiol. Rev..

[42] Suzuki T., Takahashi J., Yamamoto M. (2023). Molecular Basis of the KEAP1-NRF2 Signaling Pathway. Mol. Cells.

[43] Tare M., Sarkar A., Bedi S., Kango-Singh M., Singh A. (2016). Cullin-4 Regulates Wingless and JNK Signaling-Mediated Cell Death in the Drosophila Eye. Cell Death Dis..

[44] Huang J., Raff J.W. (2002). The Dynamic Localisation of the *Drosophila* APC/C: Evidence for the Existence of Multiple Complexes That Perform Distinct Functions and Are Differentially Localised. J. Cell Sci..

[45] Wang J., Yu H., Ma Q., Zeng P., Wu D., Hou Y., Liu X., Jia L., Sun J., Chen Y. (2021). Phase Separation of OCT4 Controls TAD Reorganization to Promote Cell Fate Transitions. Cell Stem Cell.

[46] Seif E., Kang J.J., Sasseville C., Senkovich O., Kaltashov A., Boulier E.L., Kapur I., Kim C.A., Francis N.J. (2020). Phase Separation by the Polyhomeotic Sterile Alpha Motif Compartmentalizes Polycomb Group Proteins and Enhances Their Activity. Nat. Commun..

[47] Amankwaa B., Schoborg T., Labrador M. (2022). Drosophila Insulator Proteins Exhibit In vivo Liquid–Liquid Phase Separation Properties. Life Sci. Alliance.

