# Supplementary material for: Drosophila Keap1 Proteins Assemble Nuclear Condensates in Response to Oxidative Stress
Source: Antioxidants (Basel). 2026 Jan 21;15(1):134. doi: 10.3390/antiox15010134 (PMC12838377; doi:10.3390/antiox15010134)
Supplement: Supplementary file 1 [file antioxidants-15-00134-s001.zip › dKeap1 forms condensates_supplementary.pdf]

Supplementary Materials

Drosophila Keap1 Proteins Assemble Nuclear Condensates in Response to Oxidative Stress

Guangye Ji[1,†], Bethany Cross[1,†], Thomas Killmer[1], Bee Enders[1], Emma Neidviecky[1,2], Hayden Huber[1], Grace Lynch[1], Huai Deng[1,*]

1. Department of Biology, University of Minnesota Duluth, Duluth, MN 55812
2. Current address: Department of Integrative Biology and Physiology, University of Minnesota Medical School, Minneapolis, MN 55455

* Correspondence: dengh@d.umn.edu; Tel.: +1-218-726-8459

† These authors contributed equally to this work.

Figure S1
Figure S2
Figure S3
Figure S4
Movies
Materials and Methods

Figure S1

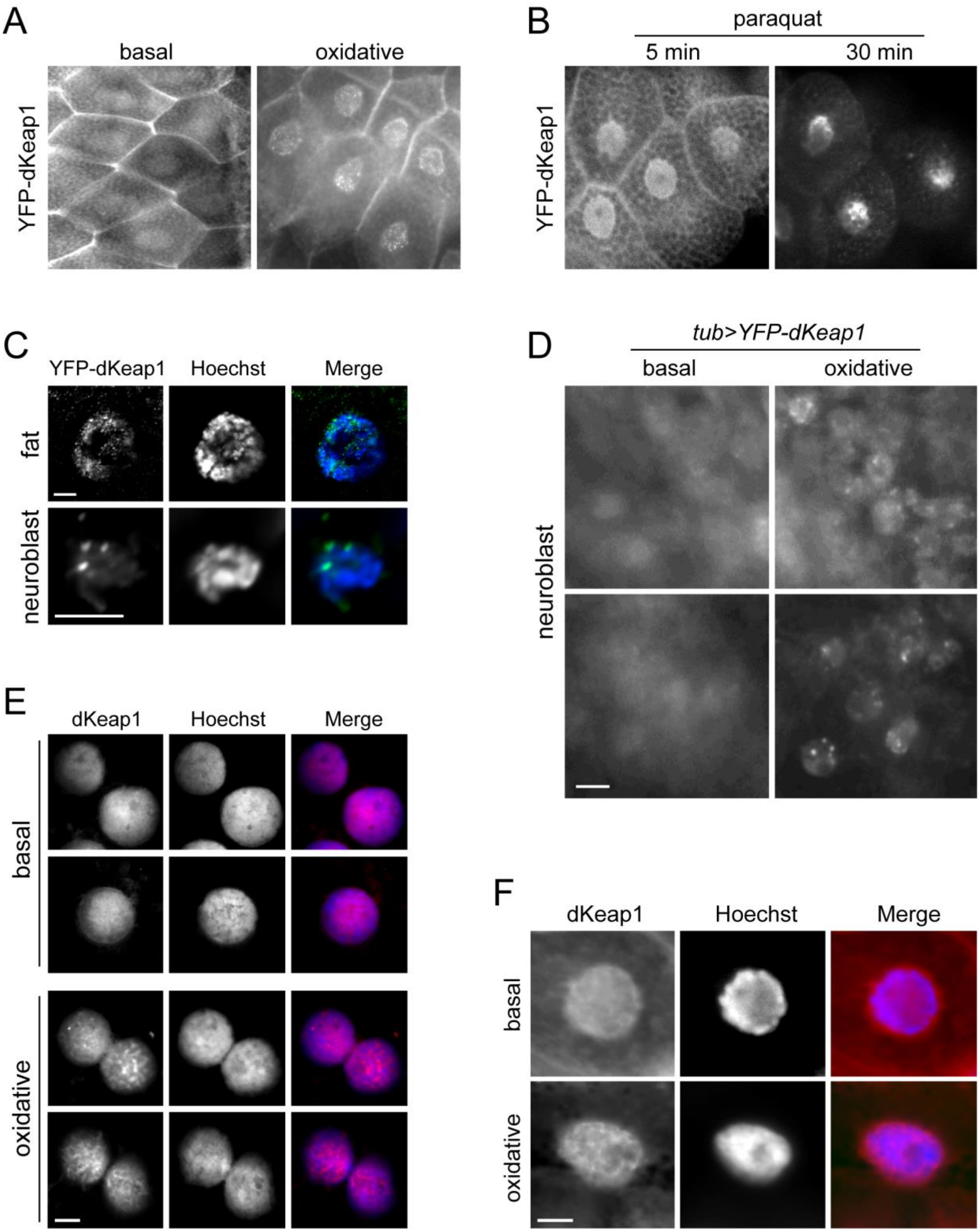

A
basal
oxidative
YFP-dKeap1
B
paraquat
5 min
30 min
YFP-dKeap1
C
YFP-dKeap1
Hoechst
Merge
fat
neuroblast
D
tub>YFP-dKeap1
basal
oxidative
neuroblast
E
dKeap1
Hoechst
Merge
basal
oxidative
F
dKeap1
Hoechst
Merge
basal
oxidative

Figure S1. dKeap1 forms nuclear foci in response to oxidative stresses

A. YFP-dKeap1 forms nuclear foci in response to oxidative stress. YFP-dKeap1 was expressed in salivary glands using *Sgs3-GAL4* driver. Live imaging assays of salivary glands were conducted immediately (basal) or after leaving in PBS for 60 minutes (oxidative).

B. Paraquat treatment induces YFP-dKeap1 nuclear foci. Salivary glands expressing YFP-dKeap1 were incubated in PBS with 50 μM paraquat for 5 or 60 minutes prior to live imaging.

C. Formation of oxidant-induced dKeap1 foci in polyploid and diploid cells. YFP-dKeap1 is expressed using *tub-GAL4* driver. Fat tissue (upper) and brain neuroblasts (lower) from L3 larvae were treated with H_2O_2 for 60 minutes and stained with anti-GFP (green) and Hoechst (blue). Scale bars: 5 μm.

D. Live imaging of oxidant-induced YFP-dKeap1 foci in neuroblast cells. YFP-dKeap1 is expressed using *tub-GAL4* driver. Neuroblasts from L3 larval brain were imaged immediately (basal) or after incubation in PBS for 60 minutes (oxidative). Scale bar: 5 μm. Robust nuclear foci formation was observed in nuclei of many oxidatively treated cells but not in untreated control cells.

E. Endogenous dKeap1 forms nuclear foci in response to oxidative stress. Wild-type salivary glands, either untreated (basal) or subjected to 60 min of oxidative stimulation, were prepared using the “smush” technique. Nuclei were stained with anti-dKeap1 (red) and Hoechst (blue). Scale bar: 10 μm.

F. Endogenous dKeap1 forms nuclear foci in response to oxidant treatment. Wild-type salivary glands, either untreated (basal) or subjected to 30 minutes of H_2O_2 treatment (oxidative), were stained with anti-dKeap1 (red) and Hoechst (blue). Scale bars: 10 μm. Enhanced dKeap1 immunostaining shown in Figure 1E and Figure S1E was achieved using a “smush” technique that releases nuclei from salivary gland cells on the slides, allowing optimized nuclear staining.

Figure S2

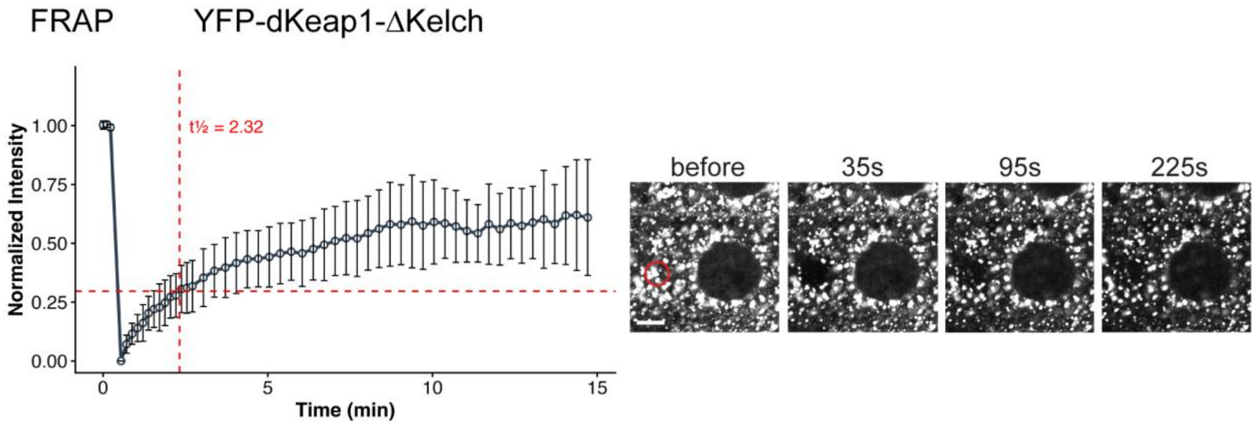

Figure S2. dKeap1-ΔKelch exhibits reduced mobility in cytoplasmic foci

FRAP analysis of cytoplasmic foci formed by YFP-dKeap1-ΔKelch. Selective cytoplasmic regions containing ΔKelch-induced foci under basal conditions were photobleached, and fluorescence recovery was quantified and plotted. Error bars indicate standard deviation from 5 independent experiments. Right: Representative images of a cell before and at the indicated time points after photobleaching. Bleached region is marked by a red circle. Scale bar: 10 μm.

Figure S3

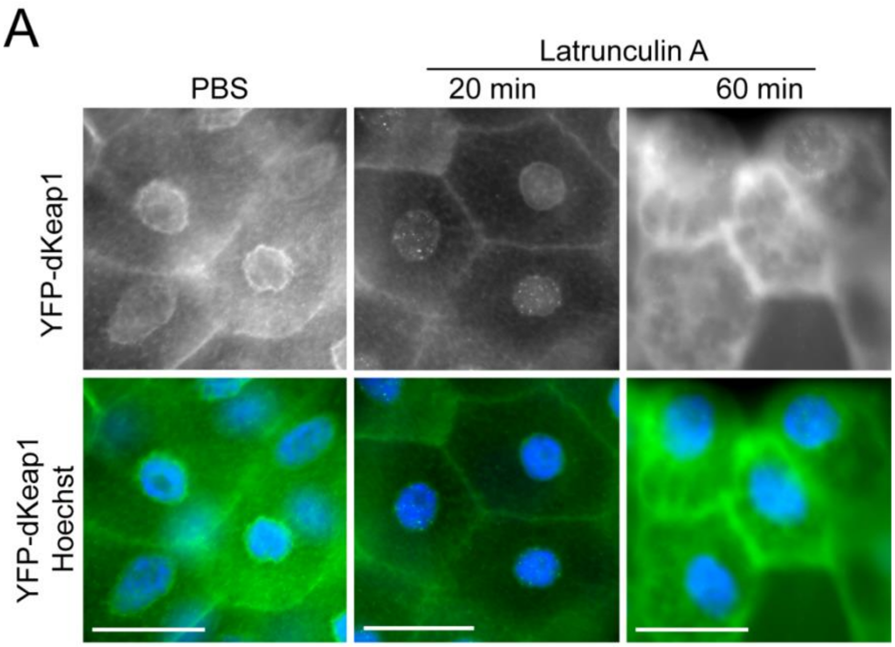

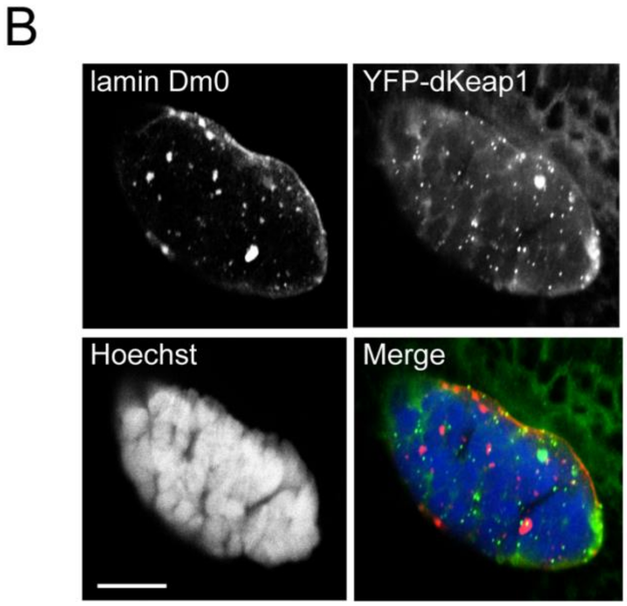

Figure S3. Actin filament and lamin are not associated with dkeap1 condensates

A. Actin filament disruption does not promote YFP-dKeap1 condensate formation.

Salivary glands expressing YFP-dKeap1 were dissected in PBS and either fixed immediately (PBS) or treated with latrunculin A for 20 or 60 minutes prior to fixation,

followed by staining with anti-GFP (green) and Hoechst (blue). Extended latrunculin A treatment disrupted salivary gland cell morphology likely due to severe loss of actin filament. However, YFP-dKeap1 remained diffusely localized and did not form foci. Scale bar: 50 μm.

B. Nuclear dKeap1 foci do not colocalize with lamin. Salivary gland cells treated with oxidative stress were stained for lamin Dm0 (red), GFP (green), and Hoechst (blue). Although overexpression of YFP-dKeap1 induces partial relocalization of lamin into the nucleoplasm, the resulting lamin signals do not overlap with YFP-dKeap1 foci, indicating that lamin proteins are not recruited into dKeap1 condensates. Scale bar: 10 μm.

Figure S4

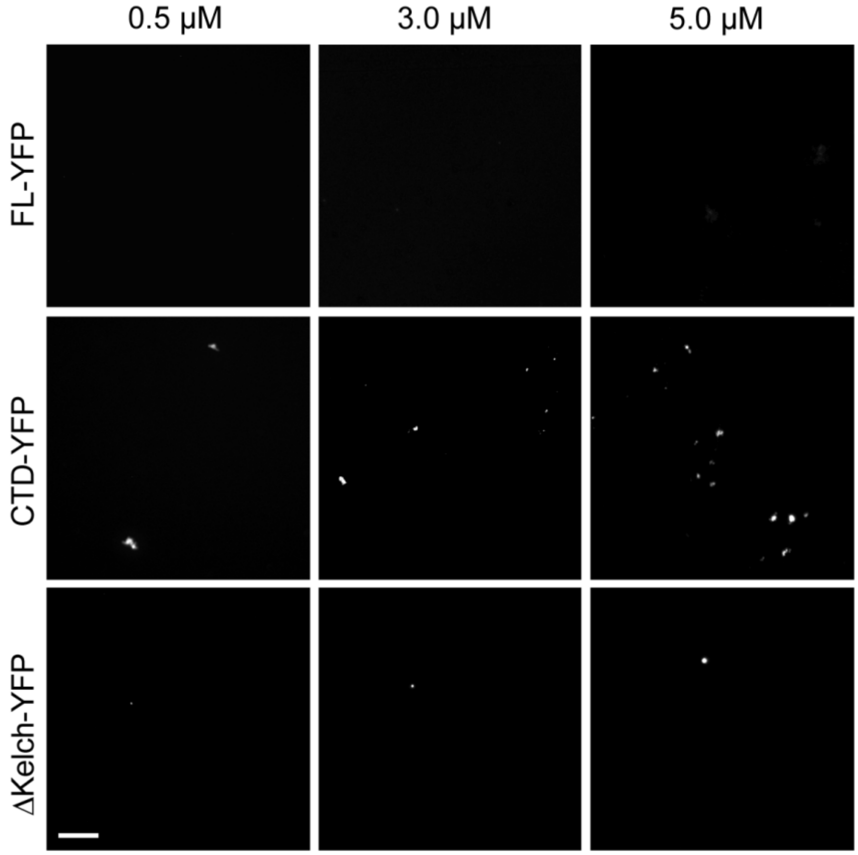

Figure S4. dKeap1-CTD undergoes LLPS in vitro

In vitro condensate formation by dKeap1 variants the absence of crowding agent. dKeap1 full length (FL), CTD, or ΔKelch fused with YFP at the C-terminus were purified. Different concentrations of these proteins indicated above were incubated in Condensate formation buffer without PEG 8000. Green fluorescence was imaged, and representative regions are shown. Full-length dKeap1 did not form condensates, whereas both the CTD and the Kelch-depleting truncation formed condensates *in vitro*, although their number was noticeably reduced compared with PEG-containing conditions (Figure 4B). This indicates that the crowding reagent is not required for LLPS and condensate formation but enhances droplet abundance. Scale bar: 10 μm.

Movies: Dynamics of YFP-dKeap1 foci in salivary gland cells.

YFP-dKeap1 and YFP-dKeap1-ΔKelch were expressed in salivary glands using the *Sgs3-GAL4* driven. Salivary glands were dissected from L3 larvae, incubated in PBS for 30 minutes, and mounted in 100 μL PBS on glass slides. A coverslip was placed on top with two edges raised using additional coverslips to prevent tissue compression. Live imaging and movie acquisition were performed using a Nikon Eclipse Ni fluorescence microscope.

S1, S2: YFP-dKeap1 formed many foci in the nucleus. Once formed, these foci were relatively stable, exhibiting only short-range movements within the nucleoplasm. No significant fusion or fission events were observed.

S3: YFP-dKeap1-ΔKelch formed numerus foci in the cytoplasm. These foci were highly stable and displayed very low mobility.

Supplemental Materials and Methods

List of Reagents

- PBS (137 mM NaCl, 2.7 mM KCl, 10 mM Na_2HPO_4, 1.8 mM KH_2PO_4, pH 7.4)
- Oxidative treatment buffer (0.5 mM H2O2 or 50 µM paraquat in PBS)
- PBST (PBS + 0.2% Triton X-100)
- Immunohistochemistry blocking and staining buffer (PBST + 1% normal goat serum)
- LB liquid medium (10 g Tryptone, 10 g NaCl, 5 g yeast extract, 950 mL H_2O)
- Lysis buffer (PBS + Roche EDTA-free protease inhibitor cocktail, 0.1 mM PMSF, 1 mg/mL lysozyme, 0.2 mg/mL DNase I)
- GST cleavage and elution buffer (supplied with PreScission Protease, APExBIO)
- Condensate formation buffer (125 mM NaCl, 50 mM Tris-HCl, pH 7.4, with or without 10% PEG 8000)

FRAP Assay

Salivary glands from L3 larvae, with or without oxidative treatment, were mounted in 100 µL PBS on glass slides. Imaging was performed on a Nikon AX-R confocal microscope using a 40× objective and a 408 nm laser for photobleaching. Bleach ROIs were approximately 6 µm in diameter, though sizes varied across experiments to match the individual dKeap1 foci.

Pre-bleach images were acquired every 6 seconds for three frames, followed by 20 seconds of bleaching. Recovery images were collected every 10 seconds for 2 minutes, then every 20 seconds for an additional 12 minutes. Fluorescence recoveries were quantified using Nikon NIS-Elements software. Global bleaching correction was performed by normalizing the bleach ROI to a reference ROI and scaling to the pre-bleach baseline as follows:

1. Background fluorescence was subtracted from both the bleach and reference ROIs.
2. To correct for global photobleaching, the background-corrected bleach ROI intensity at each time point was divided by the background-corrected reference ROI intensity.
3. The resulting values were normalized to the pre-bleach baseline, with the post-bleach minimum set to 0 and the pre-bleach average set to 1.

For each condition, 12 cells from 6 independent salivary glands were analyzed, and normalized data were pooled to generate the mean recovery curve (± SD). Processed data were imported into R (version 4.5.1) for plotting. Half-recovery times ($t_{½}$) were computed by linear interpolation at 50% of the recovery amplitude (plateau/2) using the normalized intensity values. FRAP recovery curves were generated using the ggplot2 package.

Parameter Settings for Fluorescence Microscopy

Fluorescence imaging was performed using a Nikon Eclipse Ti2 fluorescence microscope. Images were acquired with exposure times of 1 s for the EGFP channel and 2 s for the TRITC channel. Widefield illumination was provided by LED light sources set to 85% (EGFP) and 90% (TRITC). Standard Nikon filter sets were used for EGFP (Excitation 475 nm / Emission 525 nm) and TRITC (Excitation 475 nm / Emission 525).

Quantitative analysis of dKeap1 foci

Identification and quantification of dKeap1 foci was performed using ImageJ. Nuclear ROIs of dKeap1 immunosignal were automatically generated by thresholding the Hoechst signal. Within the selected nuclear ROIs, the dKeap1 signal was background-corrected. The “Find Maxima” function was then used to detect and count local intensity peaks. Each identified local peak was considered a focus. The noise tolerance parameter for Find Maxima is set to 10. To reduce duplicate counting, the points output by Find Maxima were quickly checked manually within the ROI to exclude obvious background noise or edge artifacts. Each data point represents the number of foci detected within a single cell nucleus.

The figure was generated using the R programming language, and the quantitative results are presented as scatter plots, with each point representing a cell nucleus. Open circles indicate mean values, with error bars showing mean ± SEM. Statistical significance was determined using a two-sided Wilcoxon rank-sum test.
