# Supplementary figures and images for: Drosophila Keap1 Proteins Assemble Nuclear Condensates in Response to Oxidative Stress
Source: Antioxidants (Basel). 2026 Jan 21;15(1):134. doi: 10.3390/antiox15010134 (PMC12838377; doi:10.3390/antiox15010134)

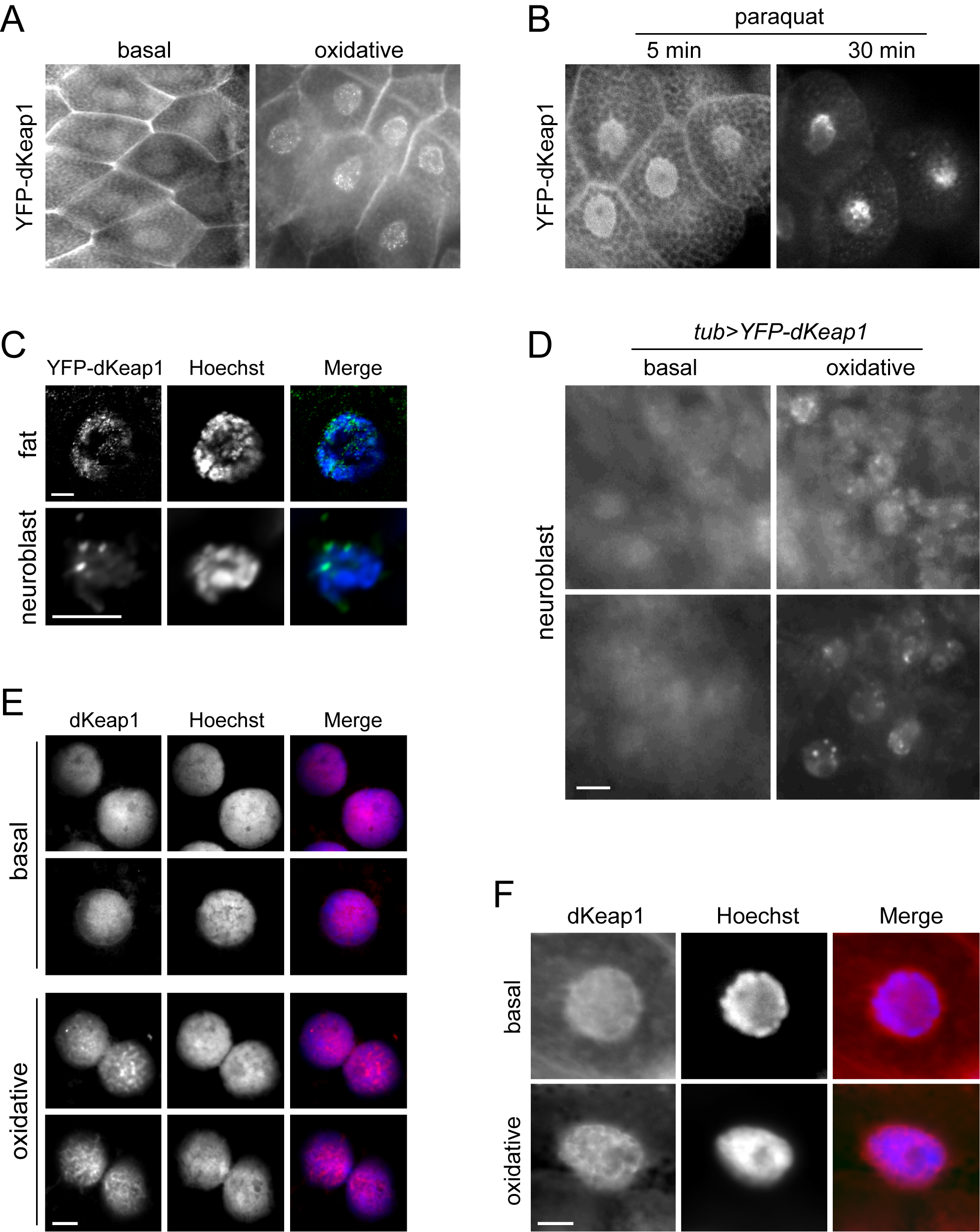

Supplement: Supplementary file 1 [file antioxidants-15-00134-s001.zip › Figure S1.tif]

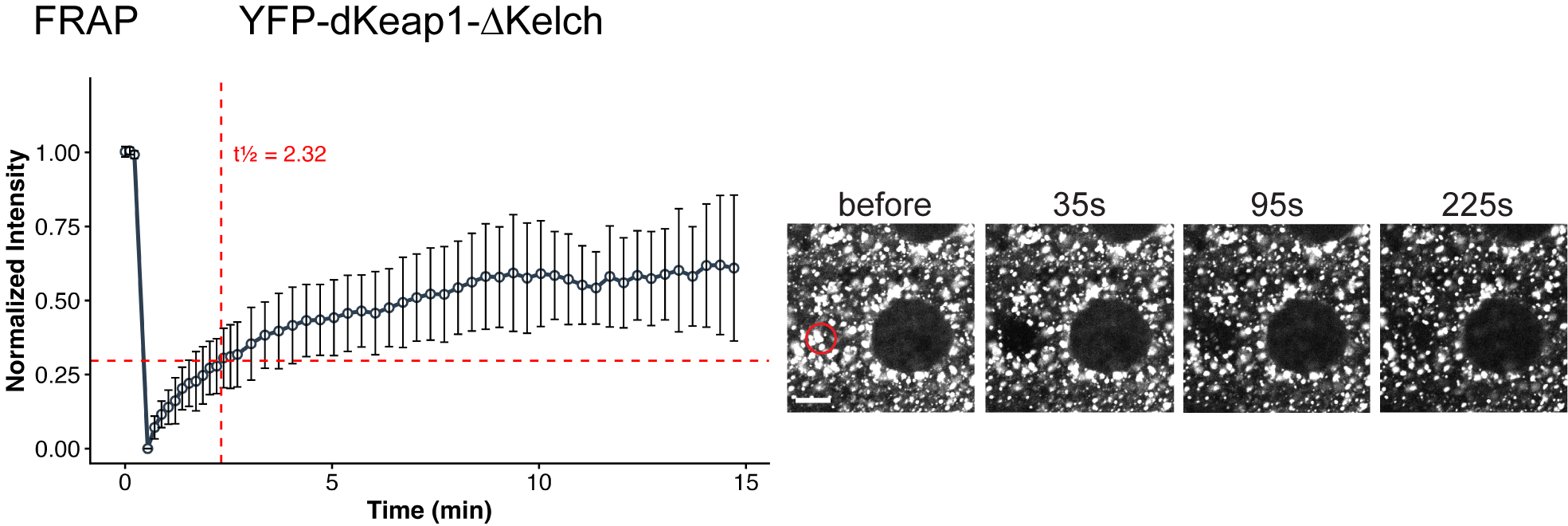

Supplement: Supplementary file 1 [file antioxidants-15-00134-s001.zip › Figure S2.tif]

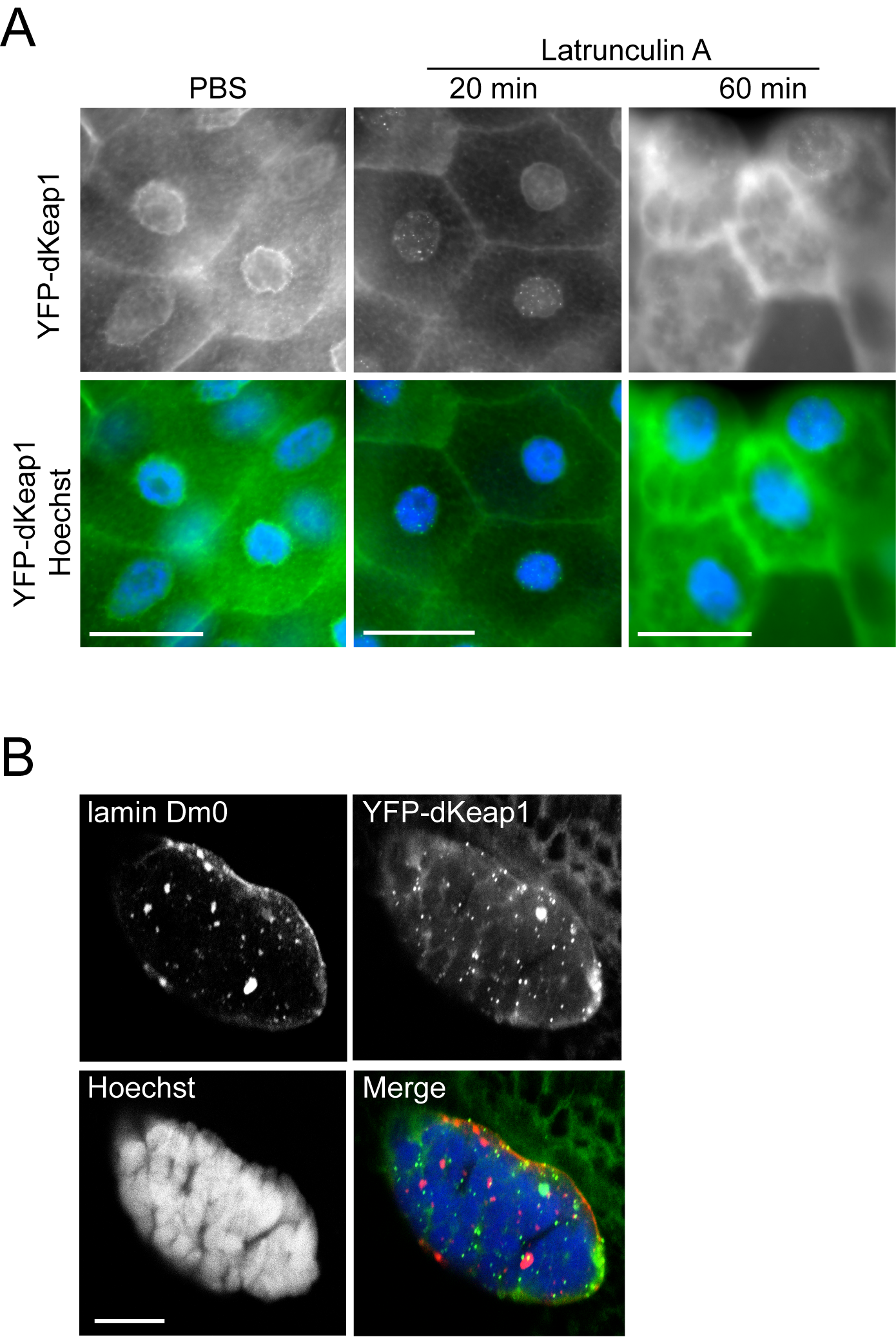

Supplement: Supplementary file 1 [file antioxidants-15-00134-s001.zip › Figure S3.tif]

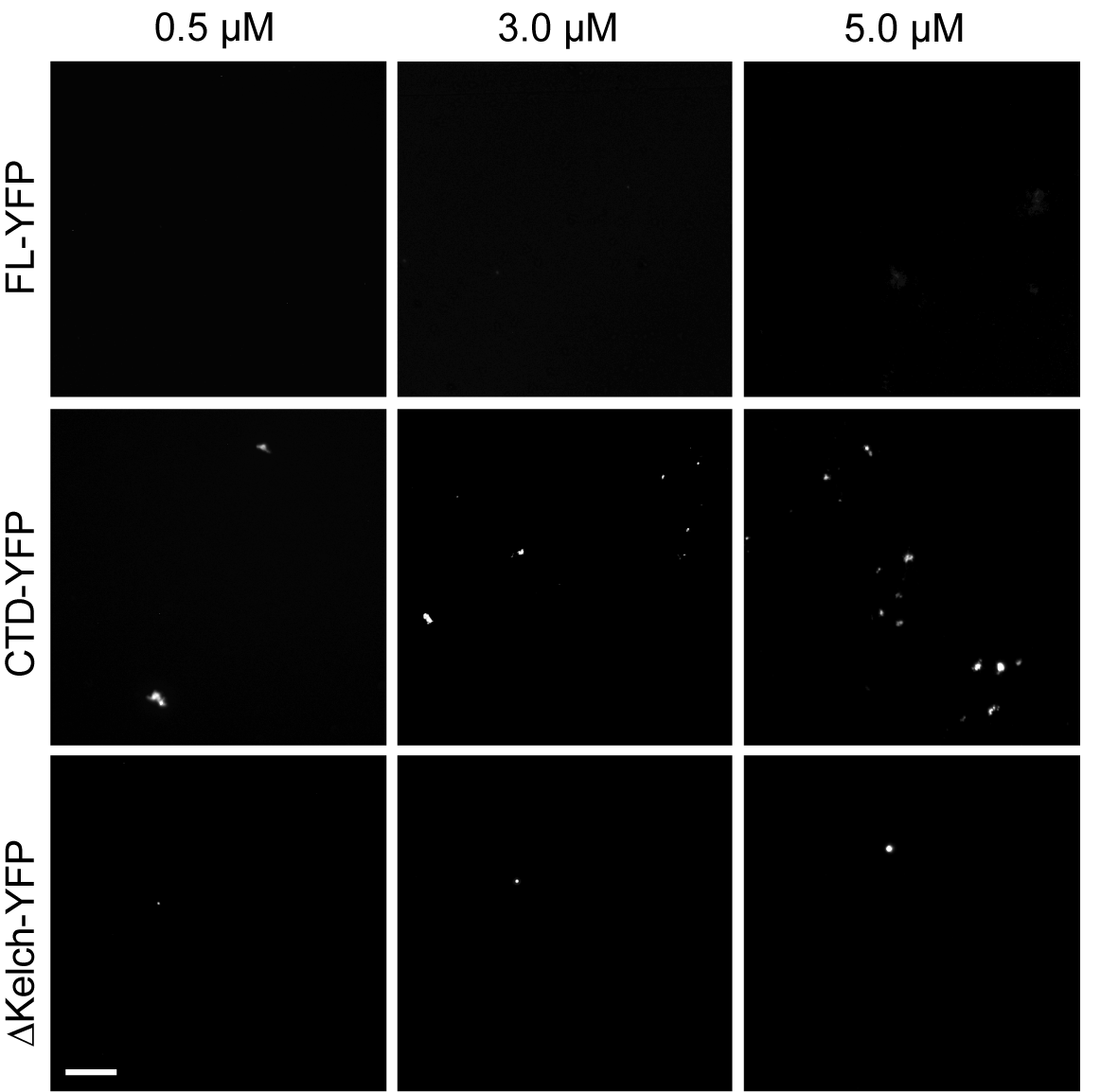

Supplement: Supplementary file 1 [file antioxidants-15-00134-s001.zip › Figure S4.tif]
